# Prognostication of Sino-Nasal Mucormycosis

**DOI:** 10.22038/IJORL.2022.58523.3021

**Published:** 2022-07

**Authors:** Prem Sagar, Namrita Mehmi, Anupam Kanodia, Avinash Shekhar Jaiswal, Rajeev Kumar, Chirom Amit Singh, Rakesh Kumar, Alok Thakar

**Affiliations:** 1 *Department of Otolaryngology & Head Neck Surgery, All India Institute of Medical Sciences, New Delhi, India.*

**Keywords:** Amphotericin, Diabetes, Mucormycosis, Sino nasal Mucormycosis, Orbital mucormycosis

## Abstract

**Introduction:**

For the purpose of prognostication of sinonasal mucormycosis, a detailed analysis of the clinical, diagnostic, therapeutic and outcome parameters has been contemplated.

**Materials and Methods:**

Retrospectively data was collected for all patients of sinonasal mucormycosis managed in a tertiary care hospital in last 5years.

**Results:**

Diabetes was the commonest comorbidity among total of 52 cases. Disease extent-wise, 16, 23 and 13 patients had sino-nasal (SN), rhino-orbital (RO) and rhino-orbito-cerebral (ROC) mucormycosis respectively. Median cumulative Amphotericin-B administered was 3.5gms and 94.2% of cases underwent surgical debridement depending on the disease extent. With a median follow-up of 18months, 67% of the patients are alive and disease free, 2% are under treatment and 29% of patients have expired. The mortality rate was 12.5% in SN, 30.5% in RO and 38.5% in ROC mucormycosis. Palatal and orbital involvement is associated with statistically significant mortality risk at one month.

**Conclusions:**

Mortality rate in sino-nasal mucormycosis can be significantly curtailed with prompt control of underlying comorbidity, aggressive medical and adequate surgical management.

## Introduction

Sino-nasal mucormycosis is an acute, fulminant and often life-threatening fungal infection which originates in the nose and paranasal sinuses. The main organisms causing mucormycosis are *Rhizopus oryzae *(most common), *Rhizopus microspores, Cunnighamella* species,* Apophysomyces* species, *Rhizomucor* species, *Mycocladus* species and *Mucor* species belonging to order mucorales (1). It commonly, but not exclusively, affects patients with diabetes mellitus (DM), blood dyscrasias, chronic kidney disease (CKD), prolonged steroid use, neutropenia, malignancies, Acquired Immunodeficiency Syndrome (AIDS), and patients on chronic deferoxamine or immunosuppressive therapy (2). The most remarkable feature of mucormycosis is vascular invasion which leads to vascular thrombosis and subsequently causes infarction and tissue necrosis. In uncontrolled diabetes, hyperglycemia and acidosis cause phagocytic dysfunction and decreased iron binding capacity and hence promote fungal growth. Clinical presentation can be confused with acute bacterial sinusitis, orbital cellulitis, acute necrotizing fibrofascitis and midline lethal granuloma. However, high index of clinical suspicion by close evaluation of symptoms and signs followed by prompt tissue diagnosis for fungus is imperative for early treatment initiation. Control or treatment of the immunosuppressing systemic illness, early initiation of anti-fungal medications and appropriate radiology to know the disease extent for planning of surgical debridement are the key to successful treatment outcome. We intend to share our experience of treating 52 patients over a period of 5 years and to different demographic and clinical factors associated with disease prognostication. 

## Materials and Methods

In a tertiary care referral center, we retrospectively reviewed the medical records of all the patients with sino-nasal mucormycosis who were managed by the department of Otorhinolaryngology from January 2013 to June 2018. The demographic, clinical, diagnostic, therapeutic and outcome parameters were reviewed for 52 patients with a microbiological diagnosis of sino-nasal mucormycosis. Also, the patients were followed-up to know their current disease status. Since it was a retrospective review and did not reveal any personal details of the patients, no consent was obtained from any of the patients. 

Patients were divided into two groups based on the survival status at the end of 4 weeks from the date of hospitalization. Group A comprised of patients who were alive at the end of 4 weeks and group B consisted of patients who succumbed to the disease. Both the groups were compared in terms of various clinical, radiological and treatment parameters. Categorical variables were compared using chi-square test or Fisher exact test and continuous numerical variables were analyzed using Mann Whitney U test or student t-test for statistical analysis. A p value of <0.05 was considered as statistically significant. 

## Results

**Fig 1:**
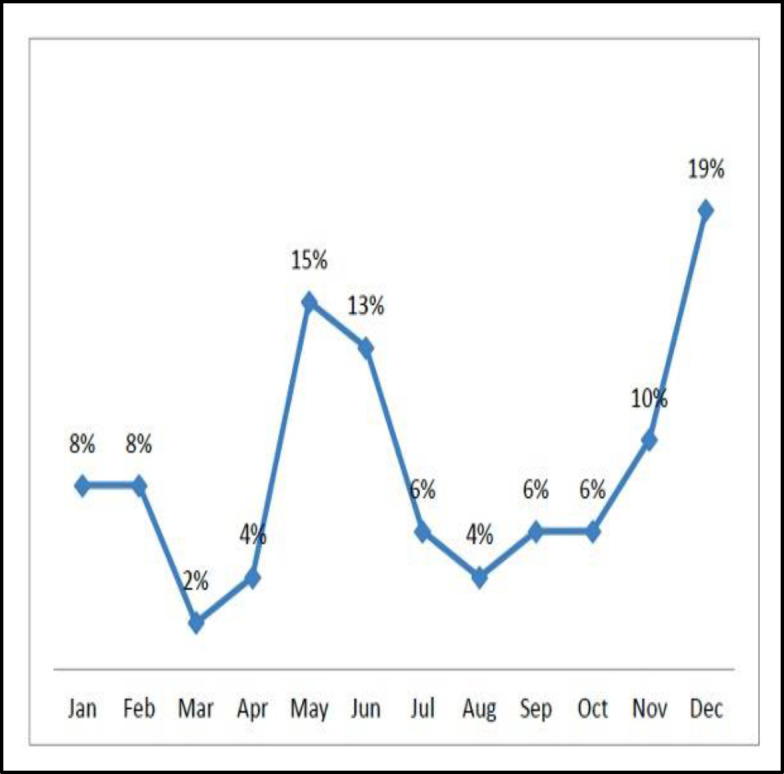
Month-wise presentation of patients based on the date of hospitalization

Out of a total of 52 patients with sino-nasal mucormycosis; 33(63%) were male and 19 (37%) were female patients with a mean age of 41.3 years and an age range of 2 to 70 years. On analyzing the seasonal pattern of presentation, we found two peaks, one in winter (19% of cases in December) and another in summer (28% of cases in May and June) as depicted in Figure 1. Most common systemic illness found in our study was diabetes in 38 (73%) cases followed by hematological malignancies in 3 (5.7%) patients and CKD in one patient. No underlying cause of immunosuppression could be ascertained in 10 (19%) patients. Out of these 10 patients, six patients were under the age of 30 years. 

The nasal smear on potassium hydroxide staining revealed broad non-septate fungal hyphae in all the cases. However, fungal culture did not grow any organism in 41 (79%) of the samples. In 21% of cases where fungal culture was positive, *Rhizopus microsporus* was grown in 11% of the samples and *Rhizopus oryzae *was isolated in remaining 10% of patients. 

**Fig 2:**
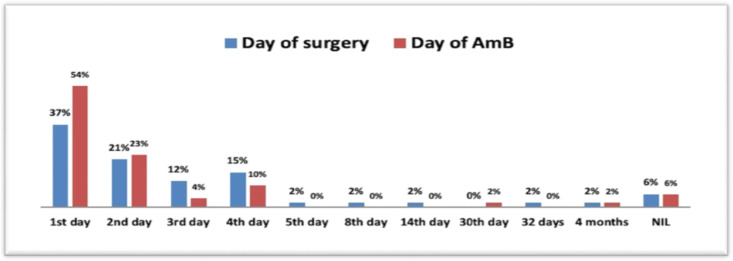
Patient distribution based on the initiation of the treatment post-hospitalization

 The mean duration of hospital stay was 39 days with a range of 2-218 days. In 90% of the patients, antifungal treatment (liposomal Amphotericin B) was started within 5 days of admission whereas in 86% of patients, surgical debridement was done within 5 days of admission (Figure 2). 

Liposomal amphotericin B was administered in a dose of 2-5 mg/kg till a cumulative dose of 4-7gms depending on radiological and clinical remission of disease. Delay in medical and surgical intervention was due to uncontrolled systemic illnesses which precluded initiation of nephrotoxic antifungals and fitness for general anesthesia. During the antifungal therapy, 15 (29%) patients developed transient hypokalemia requiring potassium supplementation and while 8 (16%) patients developed an acute kidney injury requiring temporary discontinuation of treatment. Disease extent in these patients has been described as: Sino-nasal (SN), Rhino- orbital (RO) and Rhino- orbito- cerebral (ROC). Surgical debridement was performed with the intent of maximum removal of fungal load with acceptable morbidity and the details of the surgical approach depending on the disease extent is described in Table 1. 

No surgical intervention could be performed in three patients, as two patients were unfit for general anesthesia, while one patient did not consent for surgical intervention. 

**Table 1:** Extent and route of surgical debridement performed

**Extent of surgical debridement**	**Sino-nasal**		**Additional Orbital exenteration**	**Additional Neurosurgical** **Intervention**	**None**
**Extent of** **disease (n)**	**Endoscopic**	**Open**			
Sino-nasal (16, 31%)	11	5	-	-	-
Rhino-Orbital (23, 44%)	8	13	17	-	2
Rhino-orbito cerebral (13, 25%)	7	5	10	3	1
					

During the period of hospitalization, 14 (27%) patients succumbed to disease. The remaining 38(73%) patients were discharged after completion of treatment or on medications with frequent follow-up in out-patient department after surgical debridement, control of underlying comorbidity and initiation anti-fungal medications for at least 4 weeks. The mean follow-up period was 20.7 months; median was 18 months, with a range of 6-48 months. Out of these 38 cases, 35 patients were alive and disease free, one patient had expired (uncertain cause) after remaining disease free for one year and two patients lost to follow-up. As all the 14 cases succumbed to the disease within 4 weeks of hospitalization, we divided our patients into two groups based on the survival after 4 weeks of hospitalization for analyzing the different factors associated with successful treatment outcome. 73% of the patients (n=38) survived by the end of four weeks (group A) and 27% (n=14) patients expired at the end of 4 weeks (group B). The causes of mortality in group B (n=14) patients were septic shock (n=6), acute renal failure (n=1), pulmonary thromboembolism (n=1), myocardial infarction (n=1), stroke (n=1) and miscellaneous in the remaining. 

Various clinical variables were studied among Group A and Group B patients to evaluate their association with mortality as shown in Table 2. Statistically, palatal and orbital involvement was associated with a higher mortality rate at the end of 4 weeks of hospitalization. 

**Table 2:** Analysis of clinical variables between the group A and B patients

**Parameter **	**Group A**	**Group B**	**P value**
Diabetes status (n=38)	28	10	0.870
Palatal involvement (n=21)	11	10	0.0098
Orbital involvement (n=33)	21	12	0.0431
Intracranial involvement (n=13)	9	4	0.729
Diabetic ketoacidosis (n=20)	12	8	0.11
Amphotericin started after 7 days of presentation (n=27)	20	7	0.866
Debridement done after 7 days of presentation (n=32)	24	8	0.692

The effect of surgical debridement of all the involved sites on the mortality was evaluated. Sino-nasal debridement was performed in 94.2% (n=49/52) of all patients of mucormycosis and in 100%, 91.3% and 92.3% cases of SN, RO and ROC mucormycosis cases respectively. In all the patients with orbital involvement with disease extent as RO and ROC (n=36), the effect of orbital exenteration on mortality was analyzed. We found that the mortality rate was 33.3% among these patients irrespective of whether orbital exenteration was performed of not, as shown in Table 3. Also, the effect of neurosurgical intervention on mortality rate in patients with ROC mucormycosis (n=13) was not statistically significant as mentioned in detail in Table 3. 

We also found that, the baseline blood sugar level of 250mg/dl or more in diabetic patients was found to be significantly (P value- 0.0078) associated with intracranial extent of mucormycosis with an Odds’ ratio of 7.5. 

**Table 3:** Analysis of the effect of orbital exenteration and neurosurgical debridement on the mortality rate

**Total number of cases **	**Debridement performed**		**Mortality**	**P value**
Orbital involvement n=36	Orbital exenteration			1.0
	Yes	27	9	
	No	9	3	
Intra-cranial involvement n=13	Neurosurgical intervention			0.1084
	Yes	3	2	
	No	10	1	

## Discussion

In India, the incidence of mucormycosis is 0.14 per 1000 cases of diabetics, which is about 80 times the number in developed countries (2,3). As per the current estimates, there are more than 70 million diabetics in India, and most of them have uncontrolled blood sugar levels or receive suboptimal treatment (4). The Indian climate which is mostly tropical (subtropical in part of northern India) characterized by high temperature and humidity further provides the fungus optimum conditions for growth (5). In our series of 52 cases, 38 patients had uncontrolled diabetics as the underlying predisposing factor. Many studies from different parts of India also report a significant association between diabetes and mucormycosis (6-8). Mucor infections occur due to inhalation of sporangiospores, which germinate inside the sinuses or lung alveoli in immunosuppressed hosts. Neutrophils and phagocytes form the first line of defense mechanism against this fungus, and their impaired function which is seen in uncontrolled diabetics allows the fungus to germinate and grow hyphae (9). Sino-nasal mucormycosis is the most common form of mucormycosis in diabetics, when compared with other forms like gastrointestinal, pulmonary or cutaneous mucormycosis which are comparatively common with other causes of immunosuppression (1). Apart from DM and CKD, patients suffering from conditions of iron overload, haematological malignancies, chronic corticosteroid use and organ transplant recipients are at a significantly higher risk than the rest of the population (10). 

Mignogna et al have published a case series of immunocompetent individuals, who have contracted the disease, but such cases are very rare in sino-nasal region, and the prognosis is excellent. The proposed mechanism of infection in these cases was nasal epithelial trauma and inflammation, as patients did describe vague chronic sinusitis-like symptoms (11). Roden et al also identified 176 cases with no underlying comorbidities out of 929 cases, out of which 87 developed the disease following burns, trauma or surgery (1). In our series, we could not find any common immunosuppressing illnesses in 10(19%) cases. 

We encountered the maximum cases in the month of December (winter) and May (summer) for which a definite cause could not be attributed to. A report of clustered cases of mucormycosis by Sivagnanam et al during June-August in USA suggested increased humidity as a cause (12). 

Data from Lebanon showed clustering of cases at the end of dry and warm climate (13). However, Shpitzer et al from Israel could not prove any seasonal association with the disease (14). 

In our case series, positive fungal growth rate in culture media was only 21% comprising of *Rhizopus microsporus* and *Rhizopus oryzae* in 11% and 10% of the cases respectively. Prakash et al have shown that apart from these two major agents, *Rhizopus homothallicus* and *Apophysomyces variabilis* have been found to contribute significantly to the disease burden in India (15). Bala et al identified *Rhizopus oryzae, Apophysomyces variabilis, Lichteimia ramosa, Rhizopus microsporus, Rhizopus pusillus and Apophysomyces elegans* to be the causative agents in decreasing order of frequency (6). A French review of 101 cases showed that *Rhizopus oryzae* was significantly associated with rhinocerebral forms when compared to non-rhinocerebral forms of disease (16). The low culture positivity rate for murormycosis has been attributed to the fact that the filamentous fungi get killed during the process of tissue homogenization (17).

The extent of disease in our patients ranged from sino-nasal (16 cases only) to rhino-orbito-cerebral. This distribution is observed because nasal disease causes minimal symptoms and is often ignored and managed as bacterial sinusitis. Most of the patients consult an ophthalmologist after an orbital complication develops as seen in our series where 69% of cases had orbital extent. 

A retrospective study by Sravani et al from South India reported 24 out of 30 cases of sino-nasal mucormycosis with orbital symptoms (18). However, a review of 359 cases of sinus mucormycosis by Roden et al showed that rhinocerebral mucormycosis (196 cases) was the most common extent of disease at presentation. The extent of disease at presentation depends on many factors like early access to health care, prompt suspicion of fungal etiology on initial presentation to primary physician, easy access to radiological evaluation to assess the disease extent and the severity of immune-suppression status which is directly related to rapid disease spread beyond the confines of nose and paranasal sinuses. 

Computed tomography is often non-specific for mucormycosis as only subtle mucosal thickening of the paranasal sinuses is seen in early stages. However, one can appreciate non-enhancing soft tissue in nose, paranasal sinuses, infiltrating into orbital contents and facial soft tissues, eroding palate and other adjacent bones as the disease progresses. MRI reveals involved tissue as isointense on T1-weighted and hypointense on T2-weighted images and the diseased tissue being devitalized does not enhance with contrast. Cavernous sinus thrombosis and intra-parenchymal infarcts can be seen in ROC mucormycosis on MRI(19,20). Role of radiology is to ascertain the disease extent and the diagnosis of mucormycosis is based on clinical suspicion combined with fungal staining of the affected tissue. Radiology is of importance in confirming complete disease clearance during the medical therapy and follow-up period. Management strategy to address nasal mucormycosis includes four important factors: timely diagnosis, correction or treatment of underlying systemic illnesses, prompt antifungal therapy and aggressive surgical debridement (21). A multidisciplinary approach is required in a patient of mucormycosis which includes participation of an infectious diseases internist, a microbiologist, a surgical specialist and an endocrinologist (in a case of uncontrolled diabetes). As mucormycosis causes ischemic necrosis of the involved tissue due to necrotizing arteritis, systemic antifungal agents do not penetrate the fungus-infested tissue. This necessitates radical debridement to reduce the fungal load and improves the penetration of systemically administered antifungal medication. 

An early institution of antifungal therapy with an early debridement is thought to boost the chances of patient’s survival. Liposomal amphotericin B is the antifungal of choice (22,23). Delayed diagnosis and institution of treatment has been associated with poor survival outcomes (2,24). 

However, we could not establish such an association of early (within a week of hospitalization) initiation of antifungal therapy and surgical debridement with survival as well as successful treatment outcome in our patients, possibly because of multiple confounders at play. Out of the 38 patients in group A, 36 patients were successfully treated and were disease free and two patients lost to follow-up indicating at least 69% cure rate overall. Thakar et al in their case series from our institute in 2003 reported that only three out of nine patients were alive at the end of at least 6 month follow-up and the rest had deceased (7). 

The significantly improved survival outcomes in sino-nasal mucormycosis which was considered as the deadliest infective pathology during earlier times, have been attributed to prompt diagnosis, quicker control of co-morbidities, wider availability of safe radiological investigations to assess the disease extent, newer anti-fungal medications with good safety profile which allows quicker achievement of high cumulative dose. 

Various factors affecting the survival in patients suffering from mucormycosis have been quoted in literature. A retrospective review from South Korea reported poor survival in patients with disseminated disease and improved survival in cases of pulmonary mucormycosis (21). Other studies refute this statement and suggest a poor survival in disseminated, rhinocerebral and pulmonary mucormycosis (1,25). Facial necrosis and hemiplegia were recognized as poor prognostic indicators in sino-nasal mucormycosis by Vaughan et al. (2) Yohai et al have found poor prognosis in the presence of bilateral sinus involvement, hemiplegia, underlying renal disease or leukemia and deferoxamine therapy (24). Bozorgi et al have reported female gender, advanced age, neutropenia and the presence of sinus disease to be associated with poor survival. (26). In our series, involvement of orbit and palate was associated with significantly increased mortality rate. As opposed to other studies (7,27,28). we couldn’t find any statistically significant correlation between intracranial involvement and mortality rates in our series, possibly because of relatively small sample size. We could not find any association of mortality with gender, age, presence of DKA, early institution of medical or surgical treatment and presence of other co-morbidities. However, we did find an increased prevalence of intracranial involvement in patients with uncontrolled blood sugars (>250 mg/dl). Our study has some limitations like retrospective review of data, small sample size, and non-representative patient population as ours is a tertiary care referral hospital and patients with extensive disease or severely uncontrolled underlying illnesses might have preferentially presented to us. However, following the four cornerstones of management of sino-nasal mucormycosis, we could achieve a high cure rate and significantly lower mortality rate.

## Conclusion

This retrospective data review of 52 cases of sino-nasal mucormycosis cases has revealed high successful treatment outcome rate and significantly reduced mortality rate. 

The onset of palatal and orbital involvement heralds a poor prognosis and hence, such patients should be treated aggressively. Multicentric prospective studies with a larger sample size will help us build a deeper understanding of the clinical, radiological and demographic factors affecting survival and cure rates in cases of mucormycosis. 
